# Beyond research-based literature reviews: a scoping review of methodological diversity in Swedish bachelor’s theses in nursing

**DOI:** 10.1186/s12912-025-04017-5

**Published:** 2025-10-27

**Authors:** Martin Salzmann-Erikson, Magnus Lindberg, Ann-Sofi Östlund, Marit Silén, Annika Nilsson

**Affiliations:** 1 Department of Caring Sciences, Faculty of Health and Occupational Studies, University of Gävle, Gävle, SE-801 76 Sweden; 2 Department of Public Health and Caring Sciences, Uppsala University, Uppsala, SE-751 22 Sweden

**Keywords:** Bachelor’s theses, Nursing education, Nursing undergraduates, Scoping review

## Abstract

**Background:**

In Sweden, becoming a registered nurse with a Bachelor of Science degree in nursing requires three years of full-time study, including an independent 15-credit thesis. Nursing undergraduates have limited access to ongoing research projects and clinical settings, which often prioritize master’s students and faculty-led studies. Thus, many nursing programs default to a literature-review norm, which reduces methodological diversity. This study focuses solely on non-traditional approaches, such as blog analyses, autobiographical analyses, and other innovative designs. The study seeks to disclose how these methods contribute to understanding patient experiences and advancing nursing education and research.

**Aim:**

The aim of the study was to systematically map and critically analyze the methodological and theoretical diversity within Swedish bachelor’s theses in nursing that employ alternative research methods.

**Method:**

A scoping review was conducted. Searches were performed in the DiVA portal (title-only list of 22 145 records) and in three university repositories (2 861 records), followed by an abstract-inclusive DiVA search (491 records). Screening and full-text review yielded 380 final inclusions. The national digital science archive was used to access theses completed between 2013 and 2023. Descriptive and inferential statistics were employed to analyze the data.

**Results:**

Autobiographical works were the most frequently used sources (*n* = 220), followed by blogs (*n* = 126). Dictionaries, internet forums, and combined sources were rare. Few theses used dictionaries, social media or internet forums. A descriptive research design was employed in most of the theses, and the majority focused on adults, primarily women. The theoretical content mainly covered themes related to existential issues and suffering, but several bachelor’s theses lacked a formal theoretical framework.

**Conclusion:**

Descriptive designs predominated, while exploratory and theory-integrated approaches were rare. Addressing these gaps requires pedagogical reforms that support use of diverse data sources and encourage inclusive research.

## Background

The academization of nursing education has been global in nature [1, 2], aimed at elevating the nursing profession’s status and strengthening patient safety by ensuring that care is delivered in accordance with evidence-based practice—that is, integration of the best available scientific evidence with well-established clinical expertise [3]. Initially rooted in apprenticeship models, the process has transitioned toward higher education integration [2, 4]. Key aspects include curriculum development emphasizing evidence-based practice, research methodologies, and critical thinking skills [2]. The evolution of nursing education, from a vocation to an academic discipline, can also be understood from a historical perspective through the lens of the ideologies and scientific paradigms that influenced both the curriculum and society’s views on the profession. Initially, nursing was shaped by a Christian ideological phase, where caregiving was regarded as a life mission [5]. With the advancement of medical science, nursing education transitioned into medical-ideological and medical-technological phases. By the 1980s and 1990s, a shift occurred toward a more humanistic and behavioral science perspective, known as the holistic ideological phase [6]. Simultaneously, nursing developed as a distinct scientific discipline [7]. To date, no original studies or reviews have specifically examined the methodological and theoretical diversity of nursing bachelor’s theses. In the case of Swedish nursing education, a significant transition took place with the introduction of the 1993 educational reform, which marked the beginning of the ideological academization phase, in which nurses could obtain an academic degree rather than a professional qualification, as had previously been the case [2].

In Sweden, obtaining a Bachelor of Science degree in nursing, to become a registered nurse, requires completion of 180 credits of coursework, equivalent to three years of full-time study. Within the scope of the course requirements is completion of a 15-credit independent project (thesis/degree project) [8]. Although this ordinance dates back to 1993, a significant revision was implemented in 2007 as part of the Bologna Process, which restructured the Swedish higher education system in alignment with the European Higher Education Area (EHEA) [9] The Bologna Process, established to harmonize academic degrees across Europe, aimed to promote student mobility, employability, and transparency in qualifications across 49 member states [10]. The main goal of the Bologna Process and EHEA was and still is to promote mobility, employability and competitiveness, the overall aim being to introduce systems with visible and comparable degrees. Two ways of doing this were to implement systems that divide all degrees into cycles/levels and to create a united credit system like the European Credit Transfer and Accumulations System (ECTS). In the first cycle, the basic/undergraduate/bachelor’s level, programs typically last for three years and amount to 180–240 ECTS credits. Learning outcomes were also standardized for every course. For nursing programs in Sweden, one nationally mandated outcome requires graduates to “demonstrate knowledge of the scientific basis of the field, awareness of current research and development, and an understanding of the relationship between science and proven clinical experience and its significance for professional practice” [8]. Comparable outcome-based reforms are being introduced across other EU member states [9].

Despite these structural reforms, Swedish nursing undergraduates have increasingly limited access to clinical field sites and ongoing research projects; scarce placement slots and “research-fatigued” clinical settings often prioritize master’s students and faculty-led studies^1^. Consequently, many bachelor’s programs default to a literature-review norm, which is perceived as safer to supervise and assess within tight time frames, but which reduces methodological diversity and constrains experiential learning opportunities [11, 12]. Recognizing these restrictions is essential to understanding why alternative thesis designs—when they do occur—represent a pedagogically strategic counter-move rather than a mere stylistic choice.

Previous research on nursing students’ bachelor’s theses (BTs) has encompassed a wide range of topics; however, there are few studies within the same area. Some studies have examined the process of writing a BT, from the expectations of the process [13] to the learning involved in actually writing the BT. Students have described developing skills such as organization [14], autonomous work capabilities, and critical thinking [15], as well as gaining a deeper understanding of the patient perspective and the human body. Additionally, the topic of how nurses can bring about change has been covered [12, 16].

Although writing a BT involves a high degree of autonomous work, students have stressed the importance of discussing their work with others. According to the student narratives presented in research studies, supervisors should possess strong tutorial skills and be able to motivate students [15]. Students with high self-reported self-efficacy have been found to have higher expectations of their supervisor’s knowledge than those with low self-efficacy [17]. Besides discussing the work with supervisors, discussions with peers have also been reported to be valuable [14]. Another group of studies has investigated whether there are associations between writing a BT and attitudes toward nursing research and development [18, 19] as well as evidence-based practice [14]. One study looked at experiences of gaining knowledge about providing evidence-based care when the BT was part of a clinical project [16]. Nursing students who wrote a bachelor’s thesis seemed to be more interested in, aware of and positive about research and development in nursing [18–20]. Another topic for studies on nursing students’ BTs is the choice of topic for the BT and the methods used. Regarding topics, one Swedish study found that the most common topics were experiences and managing when having an illness [21]. In comparison, another study on Danish nursing students’ BTs found that they mainly concerned nurses’ experiences of clinical practice or patients’ experiences [22]. Regarding design and methods, studies have shown different results. In one Spanish study, most BTs used quantitative methods [23], while the opposite was reported in a Swedish study. Among the reviewed BTs from Sweden, literature review was the predominant method, followed by BTs taking a qualitative approach (mostly interviews, analysis of biographies/autobiographies or blogs) and those with a quantitative approach (mostly questionnaires) [24]. One Danish study found that the nursing students used, in descending order, interviews, literature searches in databases, surveys, observation studies, document studies and mixed-methods [22]. Findings from a Spanish study showed that the most frequent types of BTs were basic research, followed by nursing care plan, research protocols and literature reviews [25]. Another, more recent Spanish study reported that students had to write their BTs in the form of a research proposal and that most of these used qualitative methods [26].

Hence, the academization of nursing education has reinforced the importance of evidence-based knowledge and research competence, rendering the BT a key component of students’ academic and professional development. While previous research has highlighted the BT’s role in fostering critical thinking and engagement with research, there is significant methodological variation across contexts. In Sweden, literature reviews and qualitative designs have been predominant, whereas other countries, such as Spain, have favored quantitative methods. However, there is an emerging body of work exploring non-traditional approaches—such as blog analyses, autobiographical studies, and other innovative methodologies—that offers new perspectives on patient experiences and nursing practice. Two recent surveys underline this international heterogeneity. A Croatian questionnaire study revealed wide variation in students’ preferred thesis types and perceived barriers [27], while a Spanish curriculum analysis showed inconsistencies in ECTS load, learning outcomes and methodological requirements across nursing programmes [25]. Yet none of these investigations examined the finished theses themselves or mapped their methodological diversity. The present study seeks to systematically identify, critically analyze, and compare alternative research methodologies employed in Swedish BTs in nursing. The study aim is to systematically map and critically analyze the methodological and theoretical diversity within Swedish BTs in nursing that employ alternative research methods. By focusing exclusively on these non-traditional approaches (such as blog analyses, autobiographical analyses, and other innovative designs), the study seeks to uncover how these methods contribute to understanding patient experiences and advancing nursing research and education.

## Methods

This scoping review adhered to Arksey and O’Malley’s five-stage framework [28], incorporating the enhancements described by Peters et al. [29], and reporting was structured according to the PRISMA-ScR checklist. A scoping review is designed to map the extent, range, and nature of research activity in a given field, identify gaps in the evidence, and clarify key concepts. We chose this approach because our aim was to comprehensively chart the diversity of methodologies used in Swedish nursing bachelor’s theses, rather than to assess the quality or synthesize results of specific studies, which is more suited to systematic reviews. Our objective was to map and describe innovative, non‐mainstream methodologies in Swedish BTs in nursing. The research questions were: what non‐traditional methodologies have been employed in Swedish nursing bachelor’s theses between 2013 and 2023, and how are they distributed across universities, data sources and analytic approaches?

### Search methods

In our initial search, we consulted a research librarian to obtain an Excel list of all BTs recorded in the Digitala Vetenskapliga Arkivet (DiVA) (English: Digital Scientific Archive) portal from January 1, 2013, to December 11, 2023. We restricted our search to theses completed between 2013 and 2023, in order to capture work produced after the full implementation of the 2007 Bologna-inspired Higher Education Ordinance, allowing an approximately five‐year transition period for curricula and assessment norms to stabilize. This ten‐year window thus reflects theses written under a consistent national degree framework, while balancing comprehensiveness and feasibility for in-depth screening. Because some universities and university colleges do not enter BTs into DiVA, we contacted the libraries at Karolinska Institutet, University of Gothenburg, Kristianstad University and Lund University, respectively, and retrieved lists of links to BTs in full text from the university repositories. Karolinska Institutet has no searchable BT repository and fiction/blog‐based BTs are not permitted, hence, that university was omitted.

#### Policy rationale and cut-off

During 2022–2023, Swedish universities prepared for revisions to the Higher Education Ordinance to comply with the EU framework for nursing education: minimum 4,600 hours with at least one-half clinical training [30]. Given the scale of curriculum and placement changes, universities jointly requested additional time; an exemption for programmes starting in 2024 was made available, and sector reports highlighted uncertainty and implementation pressures across institutions [31, 32]. In parallel, the Government announced targeted measures in 2023 to expand clinical placement capacity [33]. Because 2024–2025 constitute an implementation phase rather than a stable new normal, we predefined 2013–2023 as a policy-coherent, pre-implementation window to avoid policy confounding and preserve comparability [31, 32].

### Inclusion and exclusion criteria

To be included, a thesis had to be: a BT in a nursing program and published between 2013 and 2023 classified under nursing science, 30,305, according to the Standard for Swedish classification into research fields 2025 [34]. For detailed criteria, see Table 1.

**Table 1:** Inclusion and exclusion criteria

Inclusion criteria	Exclusion critieria
Bachelor’s thesis in a nursing programme	Research‑based literature reviews
Published between January 1, 2013 and December 11, 2023	Interview studies
Classified under nursing science (SCB code 30305)	Survey studies
Empirical work employing non‑traditional methods (e.g., narrative analysis, concept analysis, blog or podcast analysis, image analysis, social media analysis)	Master’s or specialist theses in nursing (advanced level)

### Search outcome

From the list of all records in DiVA, we found 22,145 records and 2861 from the individual university repositories, resulting in 25,006 records in total. An automated title-based filter, excluding records with Swedish keywords indicative of literature reviews (‘litteratur’, ‘litteraturöversikt’), removed 11,524 DiVA entries prior to manual screening. The remaining 13,482 BTs underwent manual search to establish whether they employed non-standardized methods or were at an advanced (master’s) level. This phase of selection was aimed at isolating studies that deviated from conventional qualitative and quantitative approaches and aligned with the inclusion criteria. Keywords such as ”narr” (an abbreviation for identifying narrative analyses) resulted in 73 theses being identified, while ”biogra” (an abbreviation for identifying biographical analyses) helped in pinpointing 85 theses. The search term ”blog” revealed 58 theses, and ”begreppsanalys” (Eng. “concept analysis”) surfaced 15 relevant works. Despite the inclusion of ”autoet” (an abbreviation for identifying autoethnographic analyses) in our search criteria, no theses were found using this term. The term ”pod” (an abbreviation for identifying podcast analyses) yielded one thesis, though it was subsequently deemed not relevant due to duplication in our manual search. Similarly, ”policy” (an abbreviation for identifying policy document analyses) and “dokumentanalys” (Eng. document analysis) led to the identification of two theses, both of which were excluded as irrelevant. Searches for theses related to social media platforms such as ”Twitter,” ”sociala med,” ”Instagram,” and ”forum” brought the total to 5, 1, and 1 thesis, respectively. Throughout this process, MSE and AN met regularly to discuss the keywords and the selected theses to reach agreement.

At the University of Gothenburg, an initial collection of 1,796 theses were identified. A total of 726 were excluded because they were not at the bachelor’s level (instead the master’s level), leaving 1,070 theses. A further 101 were excluded because they fell outside the target publication years. Nineteen BTs were excluded because they employed interview-based designs, and two were excluded for using a survey methodology. Keyword searches (*narr*,* biogr*,* blog*,* concept*,* autoe*,* policy*,* document*,* image*,* social*,* twitter*,* instagram*,* forum)* were then conducted, yielding the following hits: *narr* = 4, *biogr* = 0, *blog* = 4, *concept* = 3, and 0 hits for the remaining keywords. In total, 937 BTs were excluded because they were literature reviews. Eleven BTs remained, all of which were reviewed in full text, confirming that they met the inclusion criteria. At Kristianstad University, 549 BTs were screened. Sixty were excluded for being at the master’s level, 3 for using survey methods, 2 for employing qualitative methods that did not align with the criteria, and 483 for being literature reviews. One thesis met all inclusion criteria and was included. At Lund University, 516 BTs were reviewed. All were identified as literature reviews and thus excluded. In total, manual retrieval of 2861, excluding 2849, resulted in inclusion of 12 additional BTs.

**Fig. 1:**
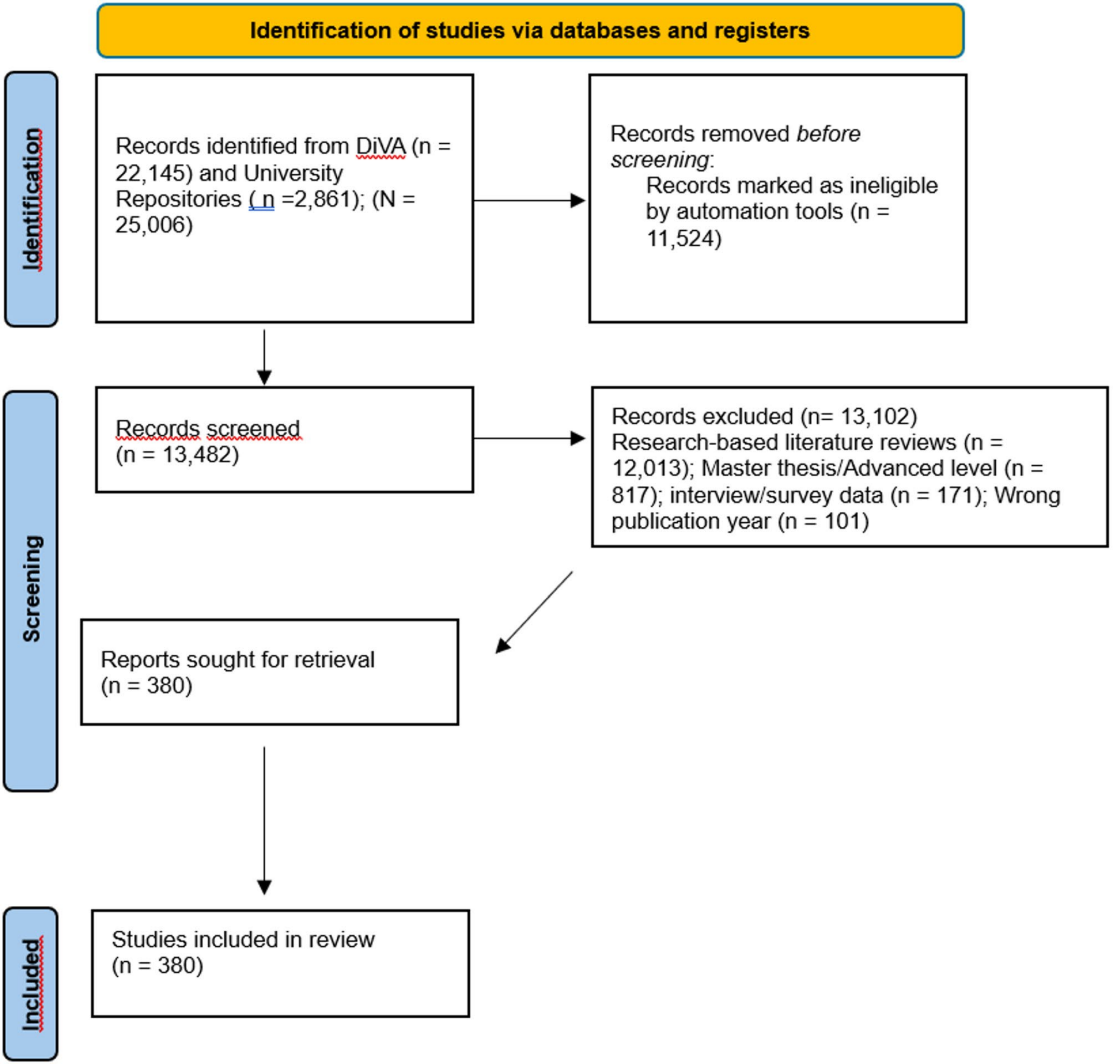
Selection strategies for the Bachelor’s thesis flow chart (PRISMA flow diagram, Page et al., 2021)

Because titles alone sometimes failed to signal non-standard designs—for example, some BTs at our own institution used blog content but indicated “litteraturstudie” in the title—we next repeated a DiVA search that allowed matching method‐related keywords in both titles and abstracts. This deeper search yielded an additional 491 thesis titles; reading abstracts excluded nine that were master’s theses, four survey studies, four interview studies, and 94 that were research-based literature reviews, resulting in 241 new inclusions from the extended, abstract‐inclusive DiVA search. Combining the 127 from the title‐only phase, the 12 from university repositories and the 241 from the abstract‐inclusive DiVA phase yielded 380 unique candidate BTs; all included in the final sample. The Prisma Flow Diagram is presented in Fig. 1 (Selection of strategies for the Bachelor’s thesis flow diagram) [35].

### Data extraction

The screening template was drafted by MSE based on early group discussions about inclusion/exclusion criteria and desired variables and moved to a shared digital platform accessible to all five authors. Author names, number of authors, publication year, thesis title, university name, aim of the thesis, research subjects, concepts or theory, sample, main focus of the thesis, core competencies, research questions, design, approach, sample selection, data source, sample size, data analysis method, depth of analysis, ethical approval were extracted and compiled into a screening template in Excel. To pilot its usability and clarity, all five authors each applied the draft template to five unique theses. Feedback from this pilot informed minor revisions to the template’s predefined options. Following the pilot, each author independently applied the finalized template to a total of 30 theses apiece (including their five pilot theses; total *n* = 150). To verify consistency, AN and MSE crosschecked 10% of ML’s, MS’s, and AÖ’s extracted data; no discrepancies in inclusion/exclusion decisions were found. Thereafter, data extraction proceeded for the remaining theses: AN extracted 75 and MSE extracted 155, completing the full sample of 380 BTs. Although Arksey and O’Malley [28] recommend dual screening, the clarity of our criteria and piloted template justified this single reviewer approach—a deviation we acknowledge as a potential limitation.

### Data analysis

Descriptive statistics (mean, standard deviation, range, skewness, kurtosis) were calculated in R (version 4.4.2) to summarize the distribution of theses across universities and to identify clusters and outliers in methodological outputs [34]. These measures enabled us to classify institutions into high, mid, and low output groups, guiding the thematic aggregation of methodological diversity. To investigate associations between data analysis methods (e.g., narrative, concept analysis) and analytic depth (manifest, latent, unspecified), we conducted Fisher’s Exact Test and computed Cramér’s V (using the lsr package). All quantitative analyses informed our narrative synthesis by highlighting significant patterns and supporting the structured presentation of methodological groupings.

## Results

A total of 380 BTs employing alternative methods were included in the present review. The distribution of authorship revealed a predominance of collaborative efforts, with the majority of theses (90.5%, *n* = 344) being authored by two students. Single-author theses were relatively uncommon, accounting for only 8.7% (*n* = 33) of the total sample. Even more uncommon were BTs with three authors (0.8%, *n* = 3).

**Fig. 2:**
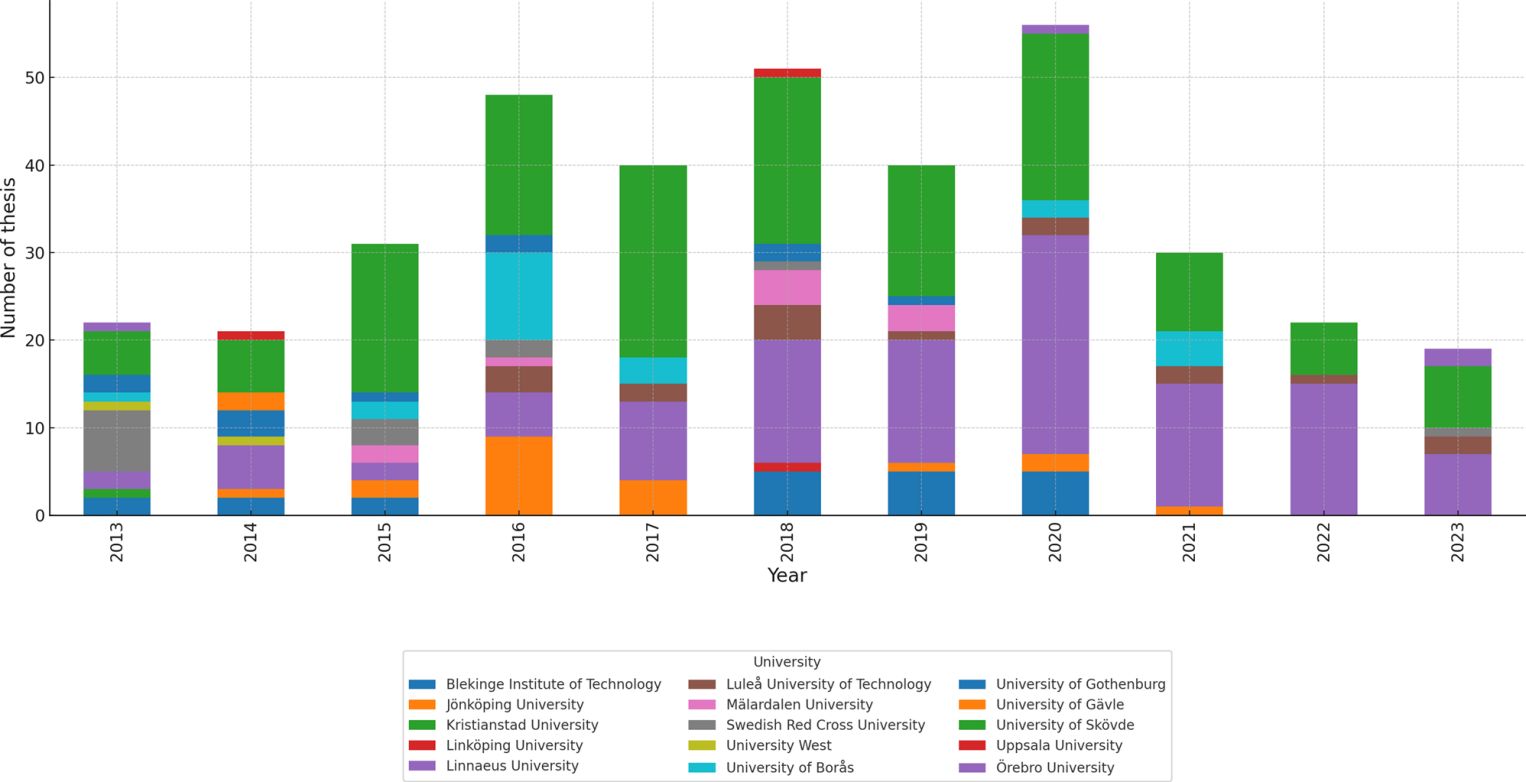
Number of theses by year and university/university college

The annual output varied, peaking in 2020 (*n* = 56), followed by 2018 (*n* = 51) and 2016 (*n* = 48); earlier years ranged from 19 to 40 theses. On average, each university contributed 25.3 BTs (SD = 42.14), with contributions ranging from 1 thesis (Kristianstad University and Linköping University) to 141 theses (University of Skövde). This variation reflects consistent contributions from some institutions, particularly the University of Skövde, whereas others produced fewer theses across the observed years. Proportional analysis indicated that the University of Skövde alone accounted for 37.1% of the total BTs produced, and Linnaeus University accounted for 29.5% (*n* = 112), highlighting the concentration of output. Other higher education institutions, including the University of Gävle, Uppsala University and University West, contributed 0.5% (*n* = 2) each. The kurtosis value of 4.50 reflects a heavy-tailed distribution, indicating that the majority of thesis production is concentrated to a few institutions with notably high output. The skewness value of 2.33 suggests a right-skewed distribution, meaning that a limited number of universities contributed to a disproportionately large number of BTs using alternative methods. This confirms that BT output is heavily concentrated among institutions with significantly higher contributions, particularly the University of Skövde. The temporal and institutional distribution of theses is illustrated in Fig. 2.

### Methodological aspects

#### Designs and research approach

The majority of the BTs using alternative methods employed a descriptive research design, with 369 (97.1%) classified as descriptive. Only 4 (1.1%) BTs were identified as using an exploratory and descriptive design, highlighting the limited engagement with exploratory methodologies within the sample. A small portion of BTs (*n* = 7, 1.8%) did not specify a research design. Regarding methodological approach, the majority of BTs (*n* = 340, 90.0%) employed an inductive approach, while a small number (*n* = 19, 5.0%) used a deductive approach, and only 2 (0.5%) employed an abductive approach. Additionally, 8 BTs (2.1%) did not specify an approach, nor could they be assigned to an approach following the full-text reading. Concerning sample selection, the majority of BTs (*n* = 339, 89.5%) employed purposive or appropriate sampling methods. Only one thesis (0.3%) reported using snowball sampling, and another thesis (0.3%) employed convenience sampling. This indicates that students predominantly selected sampling methods that enabled deliberate inclusion of participants based on specific criteria.

#### Sample populations

The majority of the BTs using alternative methods focused on adults (*n* = 187, 49.2%). Women, including both adult women and younger women, were the second most studied group, appearing in 141 theses (37.1%). In contrast, men were underrepresented (*n* = 4, 1.1%). Eleven BTs (2.9%) were family focused, while youth populations, including young women (*n* = 47, 12.4%), young adults (not gender-specific; *n* = 14, 3.7%), and teenagers (not gender-specific; *n* = 2, 0.5%), received considerably less attention. The representation of young men was particularly rare (*n* = 1, 0.3%). The high frequency of BTs with unspecified populations (*n* = 20, 5.3%) raises questions about methodological clarity in defining target populations. These patterns suggest an underlying norm in nursing research that prioritizes adult and female subjects while underrepresenting men, youth, and relational dynamics within family care contexts. This distribution reflects a tendency toward focusing research on individual experiences rather than exploring broader familial or societal caregiving structures.

#### Data sources

Regarding data sources, autobiographies and literary works emerged as the most common alternative method used in the BTs, amounting to 220 theses (57.9%). This was followed by blogs, which were used in 126 theses (33.2%), and a combination of multiple sources, appearing in 26 (6.8%). The topic of social media was cited in 3 theses (0.8%), while internet forums were referenced in 1 (0.3%), and dictionaries in 3 (0.8%). The distribution of data sources illustrates that BTs in nursing remain deeply rooted in the humanistic tradition, emphasizing narratives and personal stories. This preference for books and autobiographical content suggests that students are drawn to firsthand experiences and subjective accounts, which provide rich, qualitative insights. Meanwhile, the relatively limited use of digital platforms, such as blogs, social media, and forums, highlights an underexplored avenue for understanding contemporary patient experiences and healthcare narratives. Concerning specific data collection methods, LIBRIS (Library Information System) was the most frequently used resource, featuring 202 bibliographic BTs (53.2%), and was also used alongside other data collection methods (*n* = 42, 11.0%). Adlibris and Bokus, two online bookstores, were used less frequently, with Adlibris cited in 13 BTs (3.4%) and Bokus in 8 (2.1%). Both were employed alongside other data collection methods.

Upon further examination of the BTs with alternative methods utilizing blogs as a data source (*n* = 126), the number of included blogs ranged from 3 to 25, with a mean of 6.7 blogs per thesis (SD = 3.25). Notably, one thesis incorporated a combination of seven blogs and two video blogs (vlogs), which were collectively counted as nine blogs. Including blogs as a data source acknowledges the potential value of personal online narratives in providing nuanced, qualitative insights relevant to the research topic. Further analysis of the 220 BTs using autobiographies as a data source revealed that the number of autobiographies ranged from 1 to 70, with a mean of 5.24 (SD = 4.78) per BT. Notably, some theses incorporated multiple autobiographies from the same author, while others included a combination of autobiographies and biographical accounts. Additionally, 1 BT (0.3%) utilized internet forums as a data source, analyzing discussions from five distinct forums. This diversity of sources reflects the students’ flexible and varied approach to data collection, with a clear preference for narrative-driven and autobiographical content, providing rich qualitative insights into the lived experiences under investigation.

Four BTs (1.1%) utilized social media as a data source, reflecting an emerging approach to data collection. One thesis analyzed six Instagram accounts, focusing on visual and narrative content, while another explored 14 episodes from a podcast to capture spoken narratives and discussions. The third BT analyzed texts focused on parents’ experiences of having a child diagnosed with congenital heart disease in Sweden, 16 from Hjärtebarnsfonden’s website and 19 from Hjärtebarnsfonden’s Facebook page. Although limited in number, these examples highlight the potential of digital platforms to offer valuable perspectives and insights in nursing research.

In addition to other data sources, 25 BTs (6.6%) employed multiple data sources, 11 categorized as a ‘concept analysis.’ These theses combined a diverse array of academic and reference materials, utilizing resources such as Adlibris, LIBRIS, YouTube, and Google. They also incorporated specialized databases, including the Cumulative Index to Nursing and Allied Health Literature (CINAHL), Medical Literature Analysis and Retrieval System Online (Medline), PubMed, and the academic repository Digitala Vetenskapliga Arkivet. Furthermore, dictionaries, lexicons, and synonym dictionaries were frequently used to explore in detail the meanings and applications of key concepts. Several of these BTs expanded their scope by integrating scientific articles, textbooks, and additional media sources such as television, newspapers, and journals, providing a comprehensive contextual background. This extensive use of multiple sources reflects the depth and rigor required for concept analysis, underscoring the complexity and comprehensive nature of these investigations.

#### Ethical considerations

Of the 380 BTs included in the present review, the majority (97.4%, *n* = 370) did not report obtaining ethical approval from an external ethics committee or institutional review board. Only 10 BTs (2.6%) explicitly mentioned seeking permission from the individuals whose data were analyzed, all of which involved publicly available digital narratives such as blogs and podcasts. In these cases, the authors reached out to the content creators to request informed consent, citing ethical principles such as the Helsinki Declaration (2013) and national legislation on data protection and research ethics. These studies emphasized the importance of transparency, participant awareness, and ethical responsibility, even when working with publicly available materials. Among the remaining 370 BTs, a variety of justifications were provided for the absence of ethical approval.

#### Data analysis

The majority (*n* = 329, 86.6%) employed a manifest content analysis approach, while 48 theses (12.6%) applied latent analysis; in 3 theses (0.8%), analytical depth was unspecified. To examine whether a relationship exists between the type of data analysis method employed and the depth of analysis (manifest, latent or unspecified), a Chi-Square test was conducted. The results indicated a statistically significant association between these variables (*p* = 0.005), suggesting that the distribution of analysis depth is not random across methods. To assess the strength of this association, Cramér’s V was calculated, yielding a value of 0.182, which indicates a moderate correlation. This suggests that the relationship between analysis method and depth is meaningful, though not strong. The cross-tabulation of analysis methods and depth of analysis is presented in Table 2 (Cross-tabulation of data analysis methods and depth of analysis).

The findings highlight a clear preference for manifest analysis across all methods, particularly within qualitative content analysis (QCA), where the vast majority (247 out of 273 BTs) employed a manifest approach. Latent analysis was more frequently used in narrative analysis (16 out of 73 BTs) and concept analysis (1 out of 15 BTs), suggesting that these approaches enable a more interpretative examination of the data. The few cases in which the analytical depth was unspecified (*n* = 3, 0.8%) highlight the need for greater methodological transparency in undergraduate BTs. The significant association between analysis method and depth suggests underlying methodological patterns in thesis writing. While manifest analysis dominates due to its structured and replicable nature, latent analysis is more frequently applied in meaning-focused methods, reflecting a deeper engagement with interpretative frameworks.

**Table 2:** Cross-tabulation of data analysis methods and depth of analysis^1,2^

Data analysis method	Manifest (*n*, %)	Latent (*n*, %)	Unspecified (*n*, %)	Total (*n*, %)
Qualitative content analysis	247 (75.1)	25 (52.1)	1 (33.3)	273 (71.8)
Narrative analysis	56 (17.0)	16 (33.3)	1 (33.3)	73 (19.2)
Implication analysis	11 (3.3)	6 (12.5)	0 (0.0)	17 (4.5)
Concept analysis	13 (4.0)	1 (2.1)	1 (33.3)	15 (3.9)
Netnography	1 (0.3)	0 (0.0)	0 (0.0)	1 (0.3)
Thematic analysis	1 (0.3)	0 (0.0)	0 (0.0)	1 (0.3)
**Total**	329 (86.6)	48 (12.6)	3 (0.8)	380 (100)

^1^Chi-Square Test df = 10, *p* = 0.005

^2^Cramers V = 0.182 indicating a moderate relationship

### Nursing as a subject area

The subject matter of the included BTs highlights a strong emphasis on understanding the experiences of individuals directly affected by illness or healthcare interventions. The majority (81.8%; *n* = 311) of the theses focused on patient-centered investigations, exploring the lived experiences of individuals dealing with conditions such as cancer (primarily breast cancer) and ALS, autism, multiple sclerosis, and eating disorders. These BTs primarily aimed to provide insights into the emotional challenges and everyday realities of patients across various stages of illness. In addition to the focus on patients, a smaller proportion of theses (12.6%, *n* = 48) explored the experiences of family members and relatives, often examining the emotional toll of caregiving, grief, and loss. Studies investigating family dynamics, such as the role of parents in supporting children with chronic illnesses, were notable within this group. Concept-focused BTs (concept and implication analysis) constituted 8.4% (*n* = 32) of the sample, applying a conceptual or theoretical lens to explore key constructs within nursing. Of these, 15 were conceptual analyses. Healthcare personnel remained an underrepresented focus, with only 2.1% (*n* = 8) of the BTs examining the perspectives of professionals delivering care. A complete breakdown of sample populations is provided in Table 3. Further supporting this, an analysis of thesis titles revealed that the most frequently occurring words include ‘leva’ (live, *n* = 153, 40.3%), ‘upplevelser’ (experiences, *n* = 144, 37.9%), ‘sjukdom’ (illness, *n* = 49 12.9%), and ‘självbiografier’ (autobiographies, *n* = 79, 20.8%), reflecting an emphasis on subjective patient narratives and personal experiences of health and illness. The recurrence of terms such as ‘kvalitativ’ (qualitative, *n* = 81, 21.3%) and ‘narrativ’ (narrative, *n* = 40, 10.5%) suggests that many BTs have adopted interpretative methodologies to explore these experiences.

**Table 3:** The frequency and percentage of the sample populations in the bachelor’s thesis (*n* = 380)

Sample populations	Frequency (*n*)	Percentage (%)
Adults in general	187	49.21
Women in general	94	24.75
Young women	47	12.37
Unspecific populations	20	5.26
Young adults in general	14	3.68
Family	11	2.89
Men in general	3	0.79
Teenagers (not gender specific)	2	0.53
Elderly in general	1	0.26
Young men	1	0,26
**Total**	380	100

#### Core competencies

The distribution of core competencies within the dataset reveals a strong dominance of person-centered care, which accounts for 333 (87.6%) of the 380 BTs. This overwhelming focus underscores the centrality of individualized care and patient experiences in undergraduate nursing research. In contrast, other core competencies are markedly underrepresented: quality improvement (*n* = 4, 1.1%), team collaboration (*n* = 1, 0.3%), evidence-based practice (*n* = 1, 0.3%), and 41 BTs where the core competency was unspecified. It is noteworthy that only one BT focused on evidence-based practice, and neither informatics nor safe care, the two competences that are crucial to patient safety and digital transformation, were included in any of the theses. This suggests that undergraduate nursing students primarily conceptualize their professional role in relation to direct patient care, with less emphasis on the system-level competencies essential to the evolving demands of modern healthcare.

#### Adoption of a theoretical framework or theory

An analysis of the theoretical concepts used in the BTs using alternative methods reveals a pronounced emphasis on the negative and existential dimensions of the patient experience. The concept of “lidande” (“suffering”) appears most frequently, with 137 occurrences (36.1%), indicating that many BTs focus on the hardships and existential pain associated with illness. Close behind, “livsvärld” (“life-world”) is mentioned in 94 BTs (24.7%) and “health” appears 67 times (17.6%), suggesting that, alongside the focus on suffering, there is also an engagement with holistic, experiential perspectives. Notably, the term “none” is used in 20 theses, which implies that a substantial portion (5.3%) of the BTs do not explicitly articulate a formal theoretical framework, an observation that indicates a theoretical deficit in several theses. In addition, the theory of Sense of Coherence (SOC) is explicitly referenced in 21 BTs (5.5%), while “välbefinnande” (“wellbeing”) is noted in 45 (11.8%), indicating some recognition of resilience and positive health constructs. Overall, these results suggest that the predominant theoretical focus among the included BTs is on suffering and life-world perspectives, reflecting a strong preoccupation with the pathological and existential aspects of illness.

## Discussion

This scoping review analyzed the methodological and theoretical diversity found in Swedish BTs in nursing that used alternative research methods. Of the 380 BTs identified, most were written by pairs of students employing non-traditional methods, with literary and autobiographical works being the most common sources, followed by blogs. Few theses cited social media, dictionaries, or internet forums. The theoretical content mainly addressed suffering and existential themes, reflecting a philosophical orientation, but several BTs lacked a formal theoretical framework, highlighting a gap.

The predominance of literary and autobiographical sources indicates a preference for traditional, academically accepted materials [36, 37]. In contrast, modern platforms like blogs, social media, podcasts, and discussion forums, despite offering lived experiences, remain underused. Furthermore, the variation between higher education institutions was pronounced, with the University of Skövde alone accounting for over 37,1% and Linnaeus University 29.2% of the identified BTs, suggesting a local academic culture that supports alternative research approaches. In contrast, other higher education institutions contributed only marginally. A study by Dysthe [11] suggests that this may be due to either limited institutional support for such methods or uncertainty about how to evaluate them academically. The uneven distribution of BTs concentrated to a few higher education institutions highlights structural imbalances and raises questions about equal access to supervision, institutional support, and curriculum priorities regarding alternative research methods.

Data sources demonstrate a dual orientation: a strong humanistic perspective, emphasizing empathy and person-centered care, and a pathological focus on specific diagnoses such as cancer (e.g., breast and colorectal cancer), ALS and mental disorders. While the pathological perspective informs diagnosis, treatment, and intervention, this approach should be complemented by a humanistic orientation that sees the patient as a person, not merely a bearer of disease. Combining clinical knowledge with empathy promotes holistic care that addresses both physical health and the patient’s values, emotions, and personal preferences. This integrated approach aligns with Antonovsky´s salutogenic model [38], where salutogenic and pathogenetic perspectives are seen as complementary, rather than opposing, ways of understanding and promoting health. The frequent use of autobiographical narratives may enhance students’ understanding of the subjective experience of illness [39]. Yet the limited use of digital platforms, including social media and blogs, reveals an untapped potential to access contemporary patient narratives that reflect modern communication styles and experiences [40–42]. The predominance of manifest analysis suggests that students prioritize structured, observable content—likely mirroring training that values clarity and replicability—whereas the 48 BTs that used latent analysis show awareness of deeper interpretive approaches. BTs with an unspecified analytic mode highlight the need for greater methodological transparency.

The methodological approach in most of the BTs revealed a clear predominance of descriptive designs, with an inductive approach and purposive sampling being the most common. This indicates that students primarily focused on describing well-established phenomena rather than exploring new or under-researched areas. The low proportion of deductive (5.0%) and abductive (0.5%) approaches further highlights a lack of methodological variety and reflects a preference for simpler, more familiar research designs. This limited methodological diversity could be indicative of a hesitation to engage with more complex or uncertain topics, possibly due to insufficient exposure to advanced research methods or a lack of guidance in applying them. As noted by Meleis [43], exposure to a broader range of research methodologies and theoretical frameworks is essential in developing the ability to conduct more innovative and critical research. Strengthening the theoretical foundations of nursing education could help shift the paradigm toward a more balanced and person-centered approach.

We found that students employed a qualitative descriptive approach, typically guided by inductive reasoning. Students primarily focused on describing known phenomena rather than exploring new or under-researched areas. The limited use of exploratory designs may reflect a reluctance to engage with more uncertain or complex topics, potentially due to methodological uncertainty or, perhaps, insufficient supervisory support. Due to lack of research in this area, we cannot conclude that this pattern is solely a result of student preference. Instead, it may also be influenced by the expectations of and guidance from supervisors. As shown in a previous study of thesis assessment [44], supervisors and examiners generally agree on key quality aspects of BTs, but supervisors tend to place greater emphasis on language and formalities in addition to methodological rigor. This suggests that supervisors may encourage students to take more structured, descriptive approaches—possibly as a way to ensure clarity and that academic standards are met. Such tendencies could inadvertently steer students away from riskier, more innovative research designs, especially if they lack confidence in navigating less familiar methodologies.

Our findings align with previous research by Creswell and Poth [45] and Polit and Beck [46], who have suggested that novice researchers often favor descriptive work due to its relative simplicity compared to more complex theory building or hypothesis testing. In addition, a prevailing culture that favors established routines over innovation can further constrain both researchers’ own development and the guidance they provide students. These factors not only limit the growth of the profession, but may also reinforce existing patterns, such that students are less encouraged to challenge conventional practices or explore under-researched areas. To encourage more innovative and research-informed nursing, changes are needed at multiple levels. Integrating research into nursing education has been shown to improve students’ learning and prepare them to engage in clinical research [47]. Additionally, organizational support is critical in facilitating the adoption of new technologies and practices in nursing [48], ensuring that both students and professionals are equipped with the resources necessary to pursue innovation.

In the present study, the extensive use of purposive sampling (89.2%) suggests that students made deliberate decisions regarding the relevance and suitability of their study populations. However, a deeper analysis reveals a clear imbalance: most BTs focused on adults, particularly women and younger adult women. This skew coexists within the underlying student body: in the 2022/23 Swedish cohort, of the 5670 new nursing program entrants, 84% were women (median = 23.0 years) and 16% men (median = 24.6 years), confirming that female students predominate the discipline numerically from the outset, while men are underrepresented [49]. The scarcity of BTs specifically focused on men, three BTs, especially younger men, raises concerns about an introspective, even navel-gazing, tendency, whereby students investigate populations that mirror their own gender and social identities. Paradoxically, this selective gaze risks becoming the mirror-image of mainstream biomedical science, where male bodies have long been taken as the default and female bodies marginalized [50]. If nursing scholars reproduce an opposite but equally partial perspective, they will perpetuate a new gender-based blind spot that leaves male patients empirically under-served and undermines the promise of truly gender-responsive care. We emphasize that the over-representation of women also poses ethical questions regarding representation and equity in student-led research. However, given that the majority of nursing students are women, this pattern may reflect a form of introspective focus, where students are more inclined to investigate populations that align with their own social or gender identities. Alternatively, this tendency can be interpreted through a feminist epistemological lens as an emancipatory reaction to the long-standing predominance of male-oriented biomedical research, a critique raised by feminist scholars who have problematized positivist traditions that often marginalize women’s experiences and knowledge [51–53]. Feminist-informed research frameworks advocate for the inclusion of diverse voices and experiences, which is especially relevant in nursing, a profession grounded in holistic and person-centered care. Encouraging a broader representation in student research would not only promote methodological inclusivity, but also better reflect the complexity and diversity of clinical populations [54]. This highlights the need for nursing education to actively support more inclusive sampling strategies and critical reflection on whose voices are being prioritized or overlooked. Furthermore, the BTs focused on patients’ experiences of chronic or long-term conditions, with a strong emphasis on emotional responses and everyday challenges. This is consistent with the findings of Silén and Johansson [21], who identified patient experience and disease management as common themes, but the carer perspective, which was highlighted in their study, was largely absent from our study. Moreover, family and youth perspectives were seldom explored, and several BTs lacked a clearly defined target group. This reflects the need to broaden research focuses and include a more diverse range of populations to better reflect the multifaceted realities of nursing. In contrast, Danish studies [22] focused more on nurses’ experiences and clinical practice, which may reflect cultural or institutional priorities. This narrow methodological funnel also overlooks the dynamic interplay between students’ emotions and their sense of competence—Lundell Rudberg et al. [55] showed that positive academic emotions and feelings of ‘flow’ peak during clinical practice and thesis writing, whereas anxiety and boredom dominate in theoretical medical-science and research‐methodology courses, underscoring how richer, patient‐centred projects could better support student engagement and professional growth. One explanation is that practicing nurses seldom publish accessible firsthand narratives, because confidentiality obligations, professional ethics, and heavy workloads limit blogging or memoir writing; consequently, patient-centered sources dominate the BT data. Although understandable, this bias skews the evidence. Incorporating ethically viable alternatives such as anonymized reflective diaries, curated staff blogs, or podcasts could restore the professional perspective.

Many students treated autobiographies and blogs as consent-free “public domain” data, with only a few seeking ethics approval. This pattern was also observed in a previous investigation of Swedish BTs, which examined how students approached ethical considerations when analyzing freely available internet material [56]. However, this approach is inconsistent with the AoIR Internet Research Ethics Guidelines 3.0, which emphasize that public availability does not eliminate the need for ethical reflection and, where appropriate, informed consent [57]. Some anonymized their material, while a smaller group framed ethics as a personal responsibility. Clear program rules are needed for responsible use of public narratives. The frequent use of autobiographical sources offers rich insights, but requires strict ethical scrutiny. Netnography shows that online forums are teeming with self-appointed “experts” who mix testimony and unverified claims [58]. Swedish BTs likewise reveal that novices seldom seek consent or recognize the power gap between academic “top-dogs” and unsuspecting posters [56]. Consent, contextual sensitivity and rigorous handling of personal stories—especially those from vulnerable individuals—are therefore essential to person-centered, inclusive nursing scholarship. Students must learn about reflexive cyber-ethics and reciprocity with online contributors. Such competencies respect participant dignity, enhance BT quality and prepare future nurses for evidence-informed practice in an increasingly digital landscape.

### Relevance to practice

When undergraduates are structurally steered toward desk-based literature reviews—partly because clinical partners are over-burdened and ethical approval for primary data is harder to obtain—the predominance of descriptive, low-risk methodologies becomes less a matter of student preference and more an artefact of the educational ecology. This “methodological funnel” may safeguard minimum quality but risks dampening curiosity, limiting engagement with complex phenomena, and delivering poor “return on investment” for students who sacrifice time and tuition, yet are offered only a narrow slice of the epistemic pie, thereby keeping them from developing into truly autonomous and responsible practitioners [59]. Strategically widening access to supervised clinical micro-projects or co-production with ongoing research groups could redress this imbalance and reinvigorate thesis work, making it a transformational, not merely credentialing, experience.

## Strengths and limitations

This scoping review offered a comprehensive mapping of innovative, non-mainstream methodologies in Swedish nursing bachelor’s theses by systematically searching both the national DiVA portal and multiple university repositories. The combined use of Arksey and O’Malley’s [28] flexible five-stage framework and enhancements from Peters et al. [29], reported in accordance with the PRISMA-ScR checklist, ensured methodological rigor while allowing iterative refinement. Our broad search strategy (25 006 records screened) and detailed data charting template—piloted and refined collaboratively by all five authors—provided a transparent, reproducible approach to identifying and characterizing 380 eligible theses. Despite these strengths, the identification of sources through both DiVA and local institutional repositories required a more adaptive and less standardized search strategy than conventional bibliographic databases, potentially impacting comprehensiveness and leading to missed records. Only a single reviewer screened titles and abstracts (after initial piloting by all five authors), diverging from the dual-reviewer recommendation, which may introduce selection bias. Moreover, variability in how theses report methods and theoretical frameworks limits cross-study comparability. Our 2013–2023 cut-off was set a priori to reflect a stable policy era preceding national implementation of expanded clinical-training requirements derived from the EU framework [30]. Because 2024–2025 represent a sector-wide implementation period, with documented requests for more time and associated capacity-building initiatives, combining pre-implementation theses with transition-year theses would reduce comparability [31–33]. We therefore report 2023 as the search end date and reserve post-implementation years for a subsequent mapping.

## Conclusion

The findings of the present scoping review revealed that a limited number of universities contributed a disproportionately large number of BTs in nursing that employed alternative methods, i.e., empirical research that goes beyond the conventional paradigms of literature reviews, interview studies, and survey methodologies. This indicates that there are possibilities for universities to learn from each other regarding how BTs in nursing can be carried out. Further, the results indicated the relatively limited use of digital platforms and social media as data sources. Given the expanding amount of material available through these kinds of sources, students could be encouraged to use them if they are interested in the lived experience of a phenomenon but do not have direct access to potential participants. However, the studies that did use these kinds of sources or other publicly available texts for data collection seldom reported having sought ethical approval for the studies, providing different justifications for this. Even when such materials are publicly accessible, students must be encouraged to critically reflect on issues of consent, context, and the respectful use of personal stories, particularly when dealing with vulnerable individuals or emotionally charged content. Promoting ethical awareness in source selection and participant representation is therefore essential—not only as a methodological practice, but also as an integral component of person-centered, inclusive, and ethically grounded nursing research. A focused post-implementation mapping, once the expanded clinical-training regime has stabilized, could examine whether patterns observed for 2013–2023 persist in subsequent years.

## Data Availability

No datasets were generated or analysed during the current study.

## Footnotes

1 This observation is based on the authors’ collective experience as nursing education scholars and contributors to national academic networks discussing thesis supervision and clinical placement challenges.

## Abbreviations

BT: Bachelor Thesis
EHEA: European Higher Education Area
ECTS: European Credit Transfer and Accumulations System
DiVA: Digitala Vetenskapliga Arkivet (English: Digital Scientific Archive)
LIBRIS: Library Information System
CINAHL: Cumulative Index to Nursing and Allied Health Literature
